# Virtual interactive musculoskeletal system (VIMS) in orthopaedic research, education and clinical patient care

**DOI:** 10.1186/1749-799X-2-2

**Published:** 2007-03-08

**Authors:** Edmund YS Chao, Robert S Armiger, Hiroaki Yoshida, Jonathan Lim, Naoki Haraguchi

**Affiliations:** 1 Bjed Consulting, LLC, 9114, Filaree Ct. Corona, CA, 92883, USA; 2 Department of Bioengineering, Johns Hopkins University, Baltimore MD, 21205, USA; 3 Digital Human Center, National Institute of Advanced Industrial Science and Technology, Water Front, 3F, 2-41-6 Aomi, Koto-ku, Tokyo, 135-0064, Japan; 4 Department of Orthopaedics, Tokyo Police Hospital, Tokyo, Japan; 5 Orthopaedic Biomechanics Laboratory, Johns Hopkins University, Baltimore, Maryland, USA

## Abstract

The ability to combine physiology and engineering analyses with computer sciences has opened the door to the possibility of creating the "Virtual Human" reality. This paper presents a broad foundation for a full-featured biomechanical simulator for the human musculoskeletal system physiology. This simulation technology unites the expertise in biomechanical analysis and graphic modeling to investigate joint and connective tissue mechanics at the structural level and to visualize the results in both static and animated forms together with the model. Adaptable anatomical models including prosthetic implants and fracture fixation devices and a robust computational infrastructure for static, kinematic, kinetic, and stress analyses under varying boundary and loading conditions are incorporated on a common platform, the VIMS (Virtual Interactive Musculoskeletal System). Within this software system, a manageable database containing long bone dimensions, connective tissue material properties and a library of skeletal joint system functional activities and loading conditions are also available and they can easily be modified, updated and expanded. Application software is also available to allow end-users to perform biomechanical analyses interactively. Examples using these models and the computational algorithms in a virtual laboratory environment are used to demonstrate the utility of these unique database and simulation technology. This integrated system, model library and database will impact on orthopaedic education, basic research, device development and application, and clinical patient care related to musculoskeletal joint system reconstruction, trauma management, and rehabilitation.

## Background

The concept of the "Virtual Human" aims at the understanding of human physiology through simulation based on life-like and anatomically accurate models and data. On a grand scale, the Virtual Human will lead to an integrated system of human organ structures that explain various anatomical, physiological and behavioral symptoms and activities of a "reference human". In recent years, the explosion of science and technology, creating an overlap between the biological sciences and the engineering know-how has made the possibility of Virtual Human as a reality rather than a visionary concept. This paper introduces the development and applications of a modeling and computational software package for human musculoskeletal joint system, which will enable the execution of a wide spectrum of biomechanical analyses under simulated or experimentally measured functional environment. Therefore, this graphic modeling capability is not merely aimed for visual attraction. It is an integration of physiological simulation models coupled with computer graphics and analysis tools to determine the effects of physical, ergonomic and environmental conditions on the human body. This effort represents a trans-disciplinary collaboration among bioengineers, computer scientists, and physicians with multiple applications including medical education, basic research and clinical patient care – a precursor to the grand challenge of the "Virtual Human" concept.

This innovative concept and work in progress have long been overlooked in the field of biomedical research, but it now represents a major force among a growing number of investigators in the traditional biomechanics discipline with the added strength of new engineering technology. Engineers have been working on adapting and refining the Virtual Reality (VR) concept for model analysis and data presentation from 2D, 3D, and even 4D space through system simulation and graphic visualization. The well-known flight and vehicular simulators provide realistic environmental and human-factor conditions to train and monitor physiological responses. However, engineering aspects of VR differ from those used in the fields of entertainment and advertising. In addition to visual, tactile, and sensory requirements, bioengineering models must also satisfy the requirements of being accurate, quantitative, computational, and interactive. These fundamental premises represent the underlying objectives of the present development and application.

**Figure 1:**
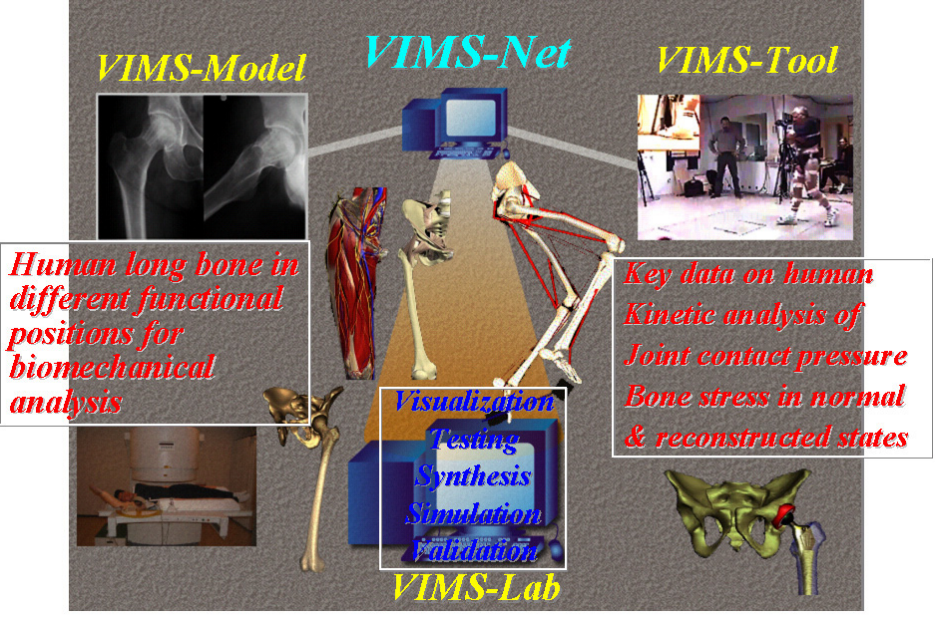
The functional flowchart and software structural platform design of the Virtual Interactive Musculoskeletal System (VIMS) and database for biomechanical analyses.

The current simulation technology described as a virtual interactive musculoskeletal system (VIMS) is a highly versatile simulation tool, providing information in an attractive, user-friendly and easy-to-understand graphic environment while allowing the theories and computational algorithms embedded in the software architecture. This musculoskeletal biomechanics simulation program is built on proprietary softwares VisModel™ and VisLab™ (Products of Engineering Animation Inc., Ames, Iowa, a subsidiary company of EDI, Huston, Texas) and other commercial utility softwares. It is divided into three highly integrated components, the "VIMS-Model"; the "VIMS-Tool" and the "VIMS-Lab" while each of them can function independently for specific application (Fig. 1). In order to handle individual variation among the normal population, homogenous, multi-dimensional and non-parametric scaling techniques will be required. The origin of the current concept and the motivation for creating a graphic-based computational model stemmed from the early work of biomechanical analyses of musculoskeletal systems and the technical problems encountered in model development and in the solution of a special class of problems [7,8,11,12,24].

Multi-body dynamic analysis of musculoskeletal system has not received the attention it deserves partially because of the modeling and analysis difficulties involved. However, the internal muscle, ligament and joint forces responsible for producing limb segment external loading and motion are still largely unknown. The redundancy of the control variables in the anatomical system and the distribution of the limb/joint forces among the tendons, ligaments, and articulating surfaces were only approximated using an optimization technique without adequate validation [15,24,25,27]. Incorporation of graphics with the model and results visualization has definite advantage but such an advance has only been attempted recently. While this proved to be a useful tool in modeling the system and in interpretation of the results, no comprehensive and in depth interactive graphics capabilities were available to execute the analyses when skeletal system is interfaced with implants or fixation devices. Buford used interactive three-dimensional line drawings in a kinematic model of the hand [5]. Later, a more attractive 3D surface model was introduced to calculate muscle-tendon paths in a biomechanical simulation environment [6]. Interactive graphical simulation software for modeling of the lower extremity has been developed [16,17]. The models presented in this paper utilized rendered and shaded three-dimensional graphics for display and allows the user to interactively set muscle paths and joint angles through a graphical interface.

A user oriented network, the "VIMS-org" (Fig. 1) will be established on the Internet to encourage close collaborations among different investigators in the musculoskeletal biomechanics community. This integrated software system and model database can impact on the learning of functional anatomy, the creation of a virtual laboratory for biomechanical analyses without the use of animals or cadaver specimens, the development of patient-specific and device-based models for preoperative planning in bone fracture management, limb lengthening, skeletal deformity correction through osteotomy, joint replacement, simulation-based intervention training using virtual instruments and environment, and the establishment of a visual feedback and biomechanics-based system for computer-aided orthopaedic surgery (CAOS) and rehabilitation.

## Graphic-based model development – "VIMS-model"

In essence, graphic-based models through simulation can bring the anatomical data to "life" through biomechanical analyses, allowing assessment of how the limb segments meet the functional demands of movement. Initially, anatomic data of the musculoskeletal system must be acquired and assembled into a model suitable for analysis and results visualization. Anatomic parameters related to joint function are quantified, including bone and soft tissue volumes, masses, and their relative orientation to one another. The ability to modify the anatomy in a model is necessary during joint function. The database contained within VIMS-Model includes generic anatomic and implant/device models, either generated or acquired, and the necessary data for musculoskeletal simulation with muscle moment arms, muscle volumes, and ligament resting lengths. These models and database are stored in suitable format that can be accessible for the computational needs to develop a single fully integrated analysis package.

### Geometric and material data acquisition

The Visible Human [36] is a set of volumetric image data of human anatomy from two cadavers serving as the main source of the generic models stored on VIMS-Model library. Boundary seeking algorithm provided by the commercial software, VisModel™ was used to map out the profile of the 3D anatomic components in order to reconstruct their surface shape volumetrically. CT data were retrieved and analyzed to build the voxels layer by layer according to preset gray level threshold to reconstruct the solid model for long bones containing different material properties and geometric irregularities. A database on isolated long bones from different populations combined with structural and material properties will be used for analysis purpose [10]. Large volume of database available in the literature and from unpublished reports will be incorporated later. This data combined with the available scaling algorithms will provide the capability of creating individual models adaptable to the generic models for biomechanical analyses.

In soft tissues, the cross-sections of these anatomic structures are outlined along their lengths, so that the centroidal lines of these tissue structures can be traced in three dimensions to define their line of action for biomechanical analyses. The muscle's physiological cross-sectional area [24] is included as an important parameter to determine muscle stress during static and dynamic activities. Muscle length and volume data are combined with their density values reported in the literature to estimate masses and moments of inertia for limb dynamic analysis. For cartilage, menisci, labrums, rotator cuff and capsules, the detailed Virtual Human dataset are used to quantify their geometry in the models mainly for computational purpose. The articular cartilage thickness is an important parameter required in the intra-articular contact stress calculation. For the other soft tissue components, their fiber bundle orientation and insertion site are important for joint loading analysis. Although these soft tissue parameters are important for biomechanical analyses, no attempt is made to graphically present them for visualization purpose due to technical difficulties and image size storage and manipulation limitation.

### Models for biomechanical analyses

In addition to musculoskeletal models, VIMS system library also contains joint replacement implant models and bone fracture fixation devices for kinematic analysis and stress/strain evaluation to study their clinical application performance through simulation studies. Several generic models available within VIMS-Model library are described here to illustrate their utility.

#### Full skeleton model

**Figure 2:**
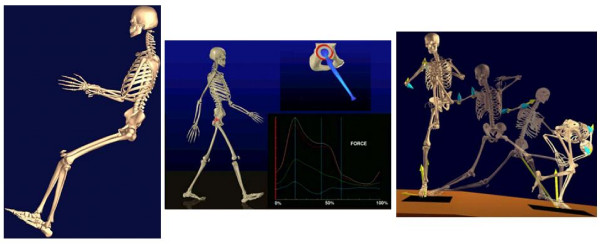
The three dimensional full-skeleton model of the human used for automobile impact study (left), gait analysis after hip replacement (middle), and the composite view of the full human skeleton to replicate baseball pitching dynamics (right). The calculated shoulder and elbow joint forces (yellow single arrow) and moments (blue double arrow) are shown together with the ground reaction force (yellow arrow) measured by a dynamic force plate for the entire cycle of pitching.

A full human skeleton model was adapted from commercial source and modified by EAI (Engineering Animation Inc., Ames, Iowa) as a general purpose surface model (Fig. 2). Local coordinate systems are imbedded in each skeletal component which can be manipulated or animated under given motion data using EAI's VisModel™ and VisLab™ software. The surface shape represented by small polygons is fixed to the local coordinate system to facilitate rigid body motion analysis and animation. This simplified model contains several integrated movable components interconnected by major anatomic joints with assumed degrees of freedom. No relative motion is permitted within the spine, trunk, hand, wrist, mid and hind foot. In spite of this limitation, this global skeletal model serves the purpose to animate human movement in normal functional activities and sports actions using measured or calculated kinematic data for visualization purpose [29].

#### Shoulder musculoskeletal model

**Figure 3:**
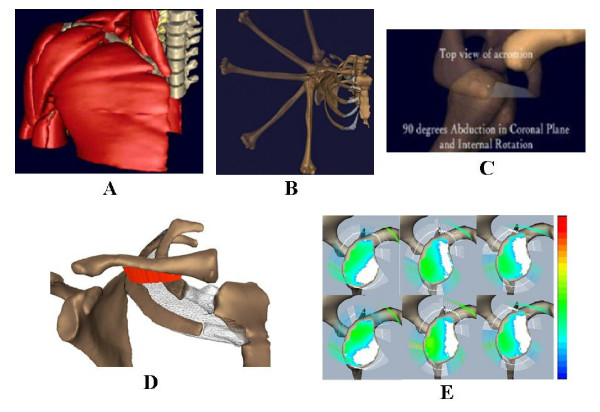
(A). A composite muscular, neurovascular and skeletal model of the shoulder visualized in a sequential manner from the superficial muscles to the underlying bony structure for anatomical studies. (B). The sequential images of a cadaver shoulder during passive elevation of the humerus in the plane of the scapula. These shoulder models were created from CT data of cadaver specimens. The kinematic data, measured by using electromagnetic "sensors" (Flock of Birds™, Ascension Technology, Colchester, VT) fixed to the humerus, scapula and clavicle and a "source" mounted on the trunk of the cadaver, was used to quantify the shoulder motion rhythm of all the bony structures involved. (C). A solid model of a cadaver shoulder highlighting the history of the closest points between the greater tuberosity and the acromioclavicular ligament during the Hawkins maneuver for impingement test. (D). The same model used to study thoracic outlet syndrome under provocative maneuver tests. The thoracic outlet area between the clavicle and the surface of the 1^st ^and 2^nd ^ribs (marked by the mesh structure) is quantified and highlighted in red color. (E). The glenoid surface model for joint contact area/stress and ligament-capsule tensile stresses study during arm elevation.

Detailed musculoskeletal models for the shoulder were constructed from cadaver specimens using their CT (for the skeleton components) and MRI (for muscles) data [18,23]. For other soft tissue details, the cryo-section images were also used. These are surface models although they provide the layered muscular, neurovascular (the brachial plexus), and all underlying skeletal structures in a composite assembly which are visible three dimensionally in a sequential and animated form (Fig. 3A). These models were used for several kinematic and functional anatomy studies (Fig. 3B–3D) and they also provided the basis for muscle joint force analysis and joint contact stress and ligament tension in activities (Fig. 3E) [28].

#### Musculoskeletal model of the pelvis and hip

**Figure 4:**
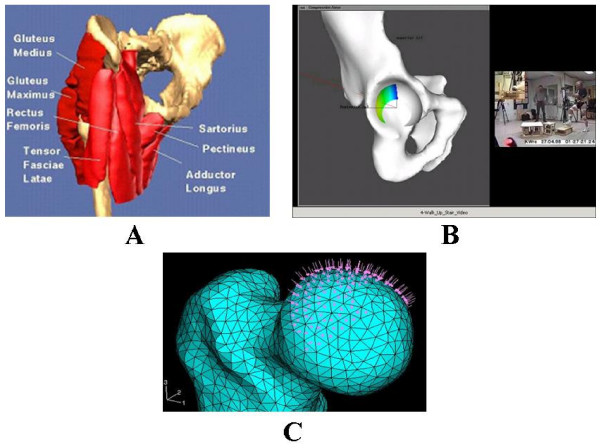
(A). The surface model of the pelvis and the proximal femur with the key muscles across the joint used for the dynamic force analysis of the hip. (B). The model used to study acetabulum contact area and stress distribution during activities of daily living involving the hip. The hip joint reaction force (arrow) and contact stress distribution at three positions during the gait cycle for the left (highlighted) leg calculated using the discrete element analysis (DEA) technique. The blue areas indicate the regions of the lowest stress while the yellow and green regions indicate the locations of higher stresses. (C). The proximal femur model used to investigate subchondral bone collapse due to osteonecrosis (OS) and femoral head reconstruction.

A composite surface model of the pelvis and all muscles across the hip joint was developed using the whole body database generated from the Johns Hopkins University, Biomechanics Laboratory and the Visible Human Dataset available on the Internet (Fig. 4A). In addition to illustrating the gross anatomy of the pelvis and the femur, this model was used to study hip joint contact stress during activities of daily living [39] (Fig. 4B). By inverting the hip joint contact stress onto the femoral head, it was also used to predict the subchondral bone collapse and investigate femoral head reconstruction due to osteonecrosis (Fig. 4C) [40].

#### Total hip replacement model

**Figure 5:**
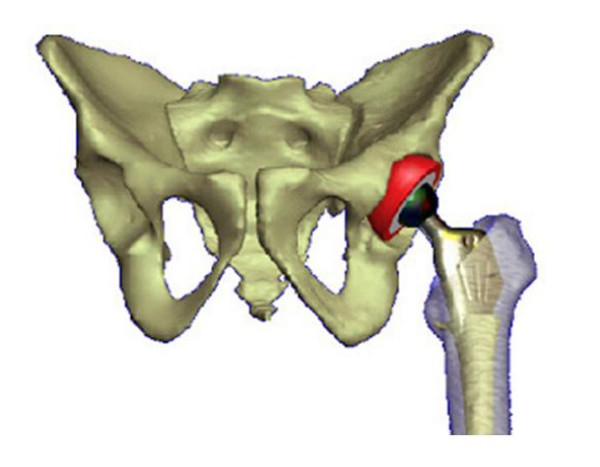
The total hip replacement model including the bone and prosthesis components used to study the effects of femoral neck design and implant placement on joint range of motion and potential dislocation.

A compounded surface and solid model for the hip joint was generated from the Visible Human Dataset to simulate total hip replacement surgery. A proximal femur/hip prosthesis model is incorporated to the pelvic model to study hip range of motion and stress distribution before and after hip replacement using different implant designs (Fig. 5) [31]. The hip implant model was developed using the CAD/CAM files from the manufacturers or taking the existing implants' plastic replicate for CT scan images. This compounded model allows both cemented and non-cemented hip replacement simulations. Joint range of motion was investigated based on acetabular component placement, joint surface wear, femoral component neck design. In addition, surgical approach and prosthesis placement were also simulated to illustrate the utility of this model.

#### Ankle joint contact stress and ligament tension model

**Figure 6:**
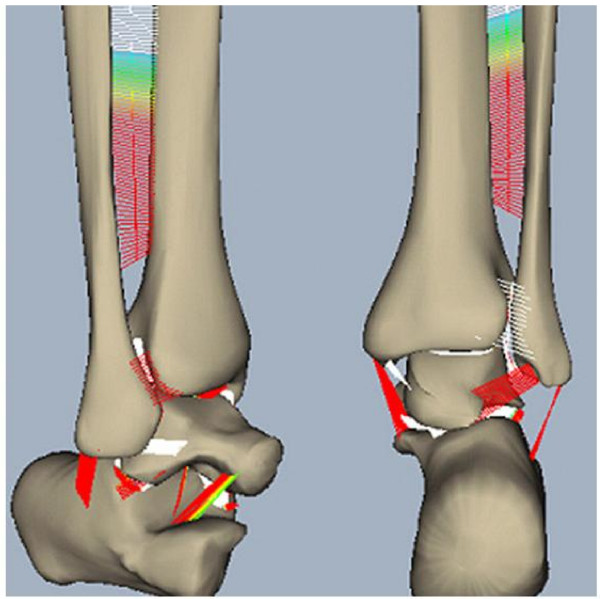
The human ankle joint model of the distal tibia, fibula, talus and calcaneus plus all the surrounding ligament connecting these bony elements.

Three-dimensional bone models of the talus, calcaneus, tibia, and fibula based on the Visible Human Dataset (National Library of Medicine) were scaled to match CT data recorded from cadaver specimens in different joint angles at 10° increments from 30° of dorsiflexion to 50° of plantar flexion covering the entire range of ankle motion during level walking (Fig. 6) [41]. Regions of potential bony contact were identified by the contour lines of the subchondral bone on each slice of the orthogonal CT sections and were then stacked to create joint contact surfaces. Rows of tensile strings for the ligaments and the interosseous membrane were inserted at the anatomical regions identified from the dissection data of the same specimen. This model was used to study ankle joint contact stress and ligament tension and to predict the location and treatment options of malleolar fracture [42]. This is the first time that the ankle normal contact and ligament stresses have been quantified using biomechanical analysis and simulation.

#### External fixator – bone fracture reduction, lengthening and osteotomy model

**Figure 7:**
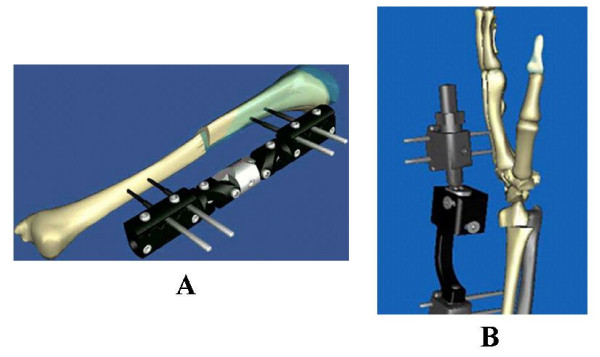
(A). The sequential exposures of the EBI Dynafix™ external fixator/tibia model illustrating the malalignment correction path by adjusting the fixator joints simultaneously in small increments. (B). The EBI DSF Dimension™ wrist fixator used to immobilize the hand relative to the forearm which could be used under the bridging type (with proximal pins in the diaphysis of the radius and distal pins in the metacarpal plus additional intermediate pin to fix the distal radial fracture fragment) and the non-bridging type (without the intermediate pin fixing the distal radial bone fragment) applications.

Three types of unilateral external fixators were modeled as solid rigid bodies of adjustable links interconnected by different joints (Fig. 7A). Any long bone or pelvis can be incorporated with the fixator forming an open or closed linkage system to study fracture reduction, bone lengthening and osteotomy adjustment through callus distraction planning using the kinematic chain theory [26]. In addition to fixator adjustibility studies, this model is now being extended to investigate fixator stiffness performance for device evaluation and design optimization. Finally, an EBI DFS Dimension Fixator™ was modeled graphically using the CAD/CAM software to demonstrate fracture reduction through fixator joint adjustment for both bridging and non-bridging applications (Fig. 7B). The parameters of a distal radius deformity were defined from the CT scans and the anterior-posterior and lateral radiographs at the fracture site. Alignment based on the bony landmarks of the radius relative to the intact contralateral side defined the deformity according to dorsal/volar translation, radial shortening and radial/ulnar translation. Radial and volar/dorsal tilts and axial rotation along the long axis of the radius described the displacement and angulation of the distal radial fragment. Because the fixator is functioning in the similar manner as a complex robotic arm, the bone-fixator system could be modeled as a multi-link closed kinematic chain [43].

There are other models stored in the VIMS "Model Library" for visualization and biomechanical analysis. Separate graphic and animation files are also archives for demonstration purpose. New models and modifications of the existing ones can be added to the library which will be updated periodically. This database is designed and managed as a "shared" resource among the VIMS users within the network described as the "VIMS.org".

### Geometric scaling of models

**Figure 8:**
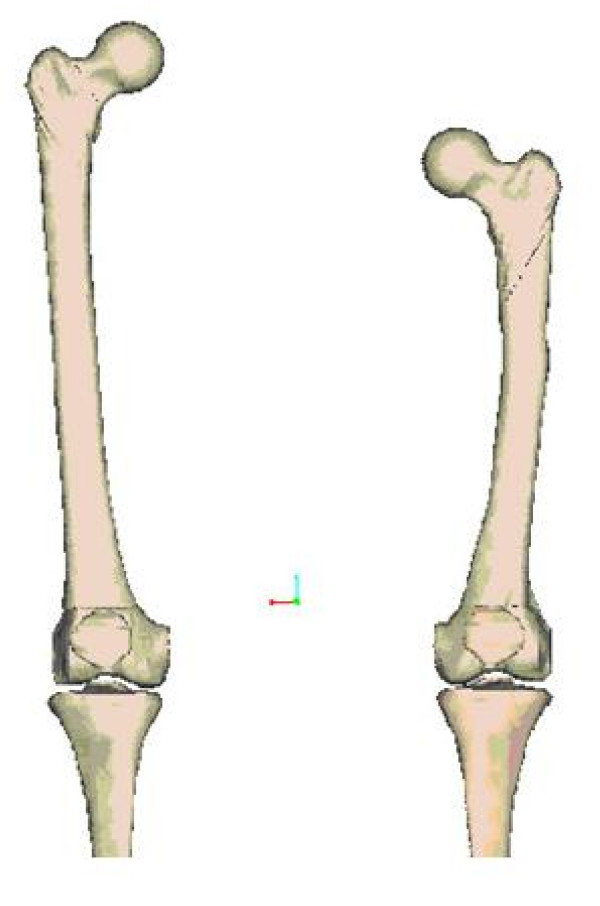
Comparison of the generic femur model and a patient-specific femur with shorter statue and a mild bowing deformity derived from the generic model using parametric scaling technique.

Nearly all models in the VIMS database are generic in nature and they were developed from the same Visible Human Dataset or the Johns Hopkins Virtual Human database. It would be impractical to utilize the same laborious process to derive an individual model for a specific person or patient for visualization and analysis purpose. To depict a patient's skeletal deformity and to perform his/her pathomechanical analysis, the specific bone and joint geometry and dimension can be derived from the generic model using the acquired x-ray or CT data in order to evaluate the biomechanical effects of the pathology and to simulate the anticipated treatment outcome based on various clinical scenarios. This method has been described as the "parametric scaling" technique in the simulation environment using custom software or commercial program such as Pro/ENGINEER™ (PTC Engineering Solutions, Parametric Technology, MA). For joint implants, spine and fracture fixation devices, scaling can be accomplished using different CAD/CAM programs. Data for each cross section of the bone can be associated with the plane or its boundary which is expressed in mathematical forms (Fig. 8). Splines used to define the cross-section boundary in each plane are modified point by point. For bone and soft tissue in the musculoskeletal system, this process is extremely difficult due to the complexity of the geometry involved.

The feature-based solid modeling technique was used in the past since the best parameters and anatomic landmarks for human appendicular and axial skeleton are largely unknown. To identify the most important parameters and quantify the range of values based on as many bones as possible should be pursued by selecting specific scaling algorithms taking the individual's age, gender, development, aesthetic and ethnic background into account. However, the VIMS-Model is intended to build a host of musculoskeletal joint generic models that can be manipulated to perform realistic biomechanical analyses on a general population or on individual patient with specific pathologic conditions. The problems associated with soft tissue scaling and graphic presentation during movement are extremely difficult to solve but they should not affect the outcome of the intended biomechanical analysis on the models subject to the known loading and motion conditions. When the precise 3D geometry of the patient's musculoskeletal anatomy and pathology is required, his/her CT and MRI data could be utilized to reconstruct the individual model with the added time and cost.

In skeletal scaling, the model must be constructed in a way that incorporates appropriate physical assumptions and mathematical approximations appropriate only for the biomechanical analyses to be performed. For structural models, computer-aided design (CAD) feature based solid modeling tools are the state of the art. While the voxel-based models with material texture or morphology incorporated are desirable, the surface models [2,33,37] are the standards for medical applications. Solid models to fit the FEM codes for stress analysis can be scaled parametrically which allow the geometry of a bone to be modified to match specific entry data. In this case, the visualization of the analysis results will be presented on more refined graphic models to enhance the appeal of complex data to both physicians and engineers.

## "VIMS-tool" for biomechanical analyses

### Kinematic analysis

In musculoskeletal systems, limb and joint motion is important to define normal functional requirements and the possible pathologic effects caused by joint diseases or neuromuscular abnormalities. Although such information could be observed or measured on living persons, no information could be derived to study the underlying skeletal movement under direct visualization. Basically, there are two types of motion, the global limb and joint motion and the local articulating surface displacement. The global motion can be quantified with fair accuracy using any of the motion analysis systems or externally mounted linkage systems. However, joint articulating surface motion is extremely difficult to measure and visualize. Therefore, the modeling and analysis capability in VIMS will be limited to global joint motion.

Joint rotations in three dimensions are expressed in terms of the familiar Eulerian Angles to facilitate musculoskeletal dynamic analyses and for movement animation. There are two most frequently used systems for Eulerian Angle definition, the "3-axes" system and the "2-axes" system. The use of the latter system is usually for the purpose of avoiding the ambiguity of rotational reference when two axes become co-liner, the "gimbal lock" phenomenon, under large range of joint motion such as in the shoulder. In two connecting skeletal segments, their relative motion from one position to another can be determined if their localized coordinate axes are defined in reference to an inertial reference frame.

Finite rotation of a limb segment is sequence dependent. However, the well-known "gyroscopic" system can be used to describe the unique Eulerian angles which will be rotational sequence independent as applied to the use of external linkage measuring device for joint motion [9,11,22]. This coordinate system was renamed as the "anatomic" axes for the knee joint [20]. It is important to note that such joint motion reference system cannot overcome the "Gimbal Lock" problem (when two of the joint rotational axes are co-linear) and since they are non-orthogonal, transformation to an orthogonal system is required for dynamic analysis.

Bone alignment correction under external fixation can be studied using rigid body kinematic analysis. When bone segments involved in fracture, osteotomy or lengthening cases are immobilized by an external fixator, the entire system can be modeled as a spatial linkage chain and studied using the movability analysis using the homogenous 4 × 4 transformation matrix [11]. Such analysis can aid to device performance evaluation, design modification, and pre-treatment planning. The skeletal-fixator system can also be regarded as a structure to study its stability behavior especially the micro-motion occurred at the bone fracture or lengthening site. The external fixator adjustibility and stiffness analyses algorithms are available in the VIMS-Tool package for specific applications in different anatomic regions. When bone lengthening or joint motion is required under external fixation, the fixator can be regarded as a robotic device to provide the ideal lengthening regime and skeletal joint motion by adjusting the components of the fixator in a predetermined fashion. This analysis program will greatly advance the technology of external fixation in orthopaedics and traumatology.

### Joint reaction forces and moments determination

A technique for quantifying the joint reaction forces and moments has been widely applied to all major joints. The algorithm for calculating the reaction forces and moments acting at these joints are based on skeletal models with inter-connecting rigid links. The mass, center of mass, and moment of inertia for the anatomic segments will be estimated or retrieved from the database in VIMS-Model. The velocity and acceleration of each link will be numerically derived from measured displacement. The joint reaction force and moment will be quantified using the Inverse Dynamics Analysis approach contained in the VIMS-Tool package [8,13,14].

### Distribution of muscle forces and joint constraints

The muscles acting about a joint will be modeled as force vectors applied along the muscle centroidal lines throughout the kinematic motion range. In VIMS-Model, the key muscles and their properties related to each joint function are documented to facilitate the dynamic analysis formulation. These muscle forces are required to balance the external forces and inertial forces acting about each joint.

Quantifying the individual muscle forces is an indeterminate problem, since there are more unknowns than equations. Therefore, optimization techniques will be combined with the equations of motion to solve for the muscle forces. The underlying assumption behind the optimization method is that the central nervous system controls muscle action by minimizing some performance criteria or cost function [1,15,25,38]. The system of equations will also be subjected to the constraint that the muscle stresses, expressed as the muscle force divided by the physiological cross-sectional area, are non-negative and bonded. Several optimization criteria are incorporated in the VIMS-Tool software and they can be refined and modified according to more up-to-date development or based on investigators' own choices.

### Intra-articular contact stress and ligament tension

The joint constraint force can be further decomposed into joint contact stresses and ligament tension using the discrete element analysis (DEA) technique [19]. This analysis technique can be modified to accommodate the mismatch in joint geometric shape and to incorporate additional soft tissues such as menisci, labrums, rotator cuff and the joint capsule. In this analysis, bones are treated as rigid bodies while the articular cartilage and the ligaments are modeled as matrices of compressive or tensile springs [21,35]. Furthermore, to satisfy the theoretical requirements of such analysis, the system must be kept in static or quasi-static equilibrium and thus allowing only infinitesimal (or virtual) displacement only in translation. The discrete element analysis (DEA) method requires less computational time than finite element analysis (FEA) techniques and it has been shown to provide equivalent results in estimating joint contact or implant/bone interface stresses [34].

Joint contact area will be determined between the two bone surfaces at each functional position. This contact area will be midway between the two bones separated by the cartilage. A compressive spring is placed on the centroid of each polygon on the concave side of the joint oriented normal to the polygon surface. Any spring that does not intercept the opposing bony surface of the joint will be eliminated from the contact area. Therefore, the joint contact area represents a subset of the joint articulating surfaces between the two bones. Ligament resting length and location are determined from the anatomic database. A series of parallel tensile springs will be used to model the ligaments or joint capsule to predict their tensile stresses in each joint position. Using the principle of minimum potential energy, the equilibrium equations describing the spring deformation are derived by applying Castigliano's theorem and the indeterminate problem can be solved using a Gauss-Jordan elimination process. The entire computational algorithm is iterative in nature since each step of joint loading under the equilibrium condition, the joint compressive springs carrying tensile load (spring length increased from its resting length before loading) or the tensile springs carrying compressive load (spring length decreased from its resting length before loading) must be removed from the system and the equilibrium analysis repeated based on the new area of joint contact and ligament cross-section. An appropriate convergence criterion will be adapted using the least-square minimization principle for the iterative process.

### Bone and implant stress analysis

Using established 3D finite element (FE) models acquired or developed, the stress and strain in the bone, ligament, implant and their interfaces can be determined using any commercial FEM codes. Special software, such as ABAQUS™ (Hibbit, Karlsson & Sorensen, Inc., Pawtucket, RI) or PATRAN™ (MacNeal-Schwiendler Corp., Los Angeles, CA) finite element code can be imported to the VIMS-Model platform to create new FE mesh using existing CT data. The size and shape of prosthesis models can be changed using the Pro/ENGINEER™ software to fit the host bone model. Interface and boundary conditions are handled by using the special element types available in the commercial codes or to be developed and incorporated to VIMS-Tool for special application. Effective post-processing software are imported and combined with the model to provide graphic presentation of the results under loading and physical activities.

## Biomechanical analyses in virtual environment – "VIMS-lab"

The applications of VIMS simulation technology to date have been limited by the availability of models and the ability to incorporate soft tissue structures in the system analysis. However, several examples are presented here to demonstrate the unlimited potential of the current technology in creating virtual laboratory environment for biomechanical analyses not possible in the past. These also help to demonstrate that the current technology is not and should not be regarded as merely a graphic-based tool for visualization purpose alone.

### Graphic animation of musculoskeletal kinematics

Graphical animation has been combined with computational analysis to animate musculoskeletal kinematics and to quantify relevant parameters related to function anatomy. Shoulder motion rhythm was investigated during passive humerus elevation (Fig. 3B) [18] and in the provocative maneuver tests used to examine shoulder impingement and the thoracic outlet syndrome (Fig. 3D) [23]. To record the kinematic data, electromagnetic sensors were used to track the motions of the humerus, scapula and clavicle in three-dimensions as the shoulders were passively manipulated. Each humerus was elevated in forward flexion, in abduction in the coronal plane, and in abduction in the scapula plane. Three provocative maneuvers used to test for shoulder impingement. To quantify the 3D motions, anatomic coordinate systems were created for the humerus, scapula, clavicle and the trunk. Geometric shapes were mapped to the graphically reconstructed bone anatomy using the iterative closest point algorithm to consistently orient the anatomic coordinate systems for each specimen [4]. The results in these studies helped to demonstrate the special utility of graphical animation to define joint coordinate systems and enhancing the interpretation of finite joint rotation results. Complex anatomical changes during skeletal movement can now be studied quantitatively under direct visualization.

### Kinematic and muscle force analysis of the shoulder

The joint reaction forces within the shoulder have been quantified for baseball pitching. The kinematic data of collegiate pitchers was collected in a motion analysis laboratory equipped with a 7-camera, 500 Hz UV-light based motion capture system (Qualisys™, Gothenburg, Sweden). Reflective markers were taped to the skin of each pitcher at the wrist, elbow, shoulder, hip, knee and ankle. A marker was also fixed to the baseball. The marker positions were digitized throughout the pitching motion and recreated on the generic full-skeleton model to animate of the pitching motion. The upper arm and forearm free-body diagram was taken at the shoulder joint for force analysis. The linear and angular accelerations of each rigid body were determined from the kinematic data. The acceleration data and the mass data from each link were used to quantify the joint reaction force and moment at the shoulder [28,29] (Fig. 2). This model is being extended for muscle and joint constraint force analysis while these forces were used to determine glenoid contact area and stress distribution during straight arm elevation in different planes (Fig. 3E).

### Hip joint pressure distribution during gait

The surface model of a pelvis and the matching left femur from the CT scan images of a male cadaver (Visible Human, National Library of Medicine) stored in VIMS-Model was used to characterize the acetabular pressure distribution during gait and in activities of daily living (Fig. 4B). The contact area of the acetabulum was determined based on the orientation of the normal vectors for each polygon on the acetabular surface mesh. The discrete element analysis (DEA) technique available in VIMS-Tool was applied to determine the contact stress distribution on the acetabulum surface under loading [19]. The contact force acting on the pelvis from the femur during gait [3] was normalized and applied to the model at five percent intervals in the stance phase of gait. The force data was transformed from the femur coordinate system to the acetabular coordinate system based on the averaged joint rotation data for normal subjects walking on a treadmill. The pelvic and femoral coordinate systems used experimentally were reproduced on the graphic model to transfer the hip joint force vector to the joint contact model.

Computer analysis of the hip joint included two stages. At the first stage, the shapes of the femoral head and the acetabulum were created from the Visible Human dataset [1] and the potential contact area for an individual was established from his/her anteroposterior radiograph data [2]. The cartilage between the acetabulum and the femoral head was represented by 4000 'equivalent' unilateral springs. In order to find the joint pressure distribution the acetabulum and the femoral head were assumed to be rigid bodies. The loads at the joint were chosen as the hip contact forces during ADL [3]. The pressure distribution was obtained by using the discrete element analysis (DEA). At the second stage, the hip pressure obtained from the DEA was inverted to a distributed load on the femoral head for the subsequent finite element (FE) analysis.

The spatial finite element model used continuum brick elements for the cancellous bone and thin shell elements for the cortical bone. The nodal points of the contacting brick and shell elements were properly adjusted. The femoral head was rigidly fixed at the plane separating it from the femoral neck. The necrotic area was considered as a cone with the base angle of 2p/3 radian. ABAQUS™ software (Hibbit, Karlsson & Sorensen, Inc., Pawtucket, RI) was utilized for the eigenvalue buckling (instability) analysis of the cortical shell under the normal and necrotic conditions.

Two Eigenvalue buckling analyses were performed under the AVN conditions of degrading elastic properties of the femoral head [40]. First, the Young modulus of the cortical shell was chosen to be equal 1.0 GPa and the critical pressure was computed for the varying Young modulus of the cancellous bone: the left diagram. Second, the Young modulus of the cancellous bone was chosen to be equal 1.0 MPa and the critical pressure was computed for the varying Young modulus of the cortical shell: the right diagram which may lead to femoral head collapse (Fig. 4C). When the normal femoral head was considered where the Young modulus was chosen to be equal 10.0 GPa and 1.0 GPa for the cortical and cancellous bone accordingly, negative critical pressure was obtained reflecting the strong compressive strength to sustain any of the normal loading applying to the hip.

### Ankle joint contact stress and ligament tension during stance phase of gait

Joint articular contact and ligament loading were explored using the DEA technique by establishing a region of elastic elements between rigid bodies representing bones. Articular cartilage was represented by compressive springs and ligamentous tissue was modeled using tensile springs. Three-dimensional bone models of the talus, calcaneus, tibia, and fibula based on the Visible Human Dataset (National Library of Medicine) were scaled to match CT data recorded principally for this study of a cadaver in different flexion angles at 10° increments from 30° of dorsiflexion to 50° of plantar flexion which covered the entire range of ankle motion during level walking. Regions of potential bony contact were identified by the contour lines of the subchondral bone on each slice of the orthogonal CT sections and were then stacked to create a contact surface. The contact surfaces were subdivided into approximately 10,000 triangular mesh elements to place the unidirectional compressive springs. Rows of tensile springs for the ligaments and the interosseous membrane were inserted at anatomical positions as identified from dissection data of the same specimen. The stiffness of the springs was determined from previous data.

We applied physiological loads approximating that during normal walking based on previously published data [1] for an 80 kg subject. Constraint forces through the ankle joint were used to calculate the deformation of the spring element system once an equilibrium state was achieved. The model was evaluated at discrete frames during the stance phase of gait to study the relationship between ankle position and joint loading on the contact mechanics characteristics and the loading of individual ligaments (Fig. 6) [41].

**Figure 9:**
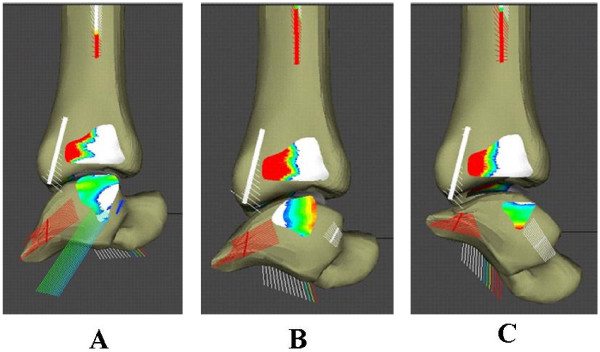
Ankle contact stress distribution and ligament tension in the tibiotalar and talofibular joints during the stance phase of gait. (A) early stance, ankle in plantar-flexed position, (B) mid-stance, ankle in neutral position, (C) late stance, ankle in dorsi-flexed position.

**Figure 10:**
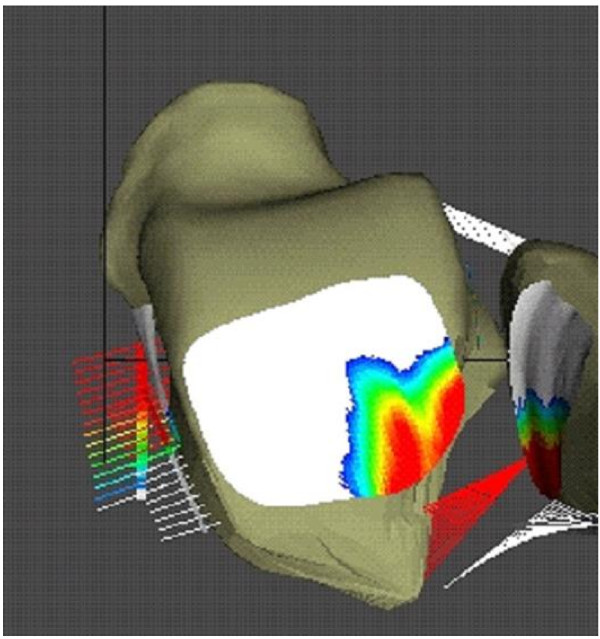
Contact pressure distribution at the tibial platfond loaded in ankle dorsi-flexed position.

Contact characteristics and ligament tensions associated with the normal ankle joint during the walking cycle are shown (Fig. 9). As anticipated, the major ankle joint loading during the stance phase is through the articular surface of the joint. However, the posterior tibiofibular ligament (especially the inferior branch) was loaded during the heel strike and toe off periods of the stance phase. Loading of the medial malleolus was observed only near the mid-stance to heel off frames when dorsiflexion was involved. The tibio-talar articulation showed full congruency through the majority of the stance phase with peak pressure developing anteriorly towards the toe-off frame. As the flexion angle of the ankle joint changed from dorsiflexion to plantar flexion, the posterior malleolar contact pressure increased (Fig. 10). Hence, when patients with malleolar fractures and treated with cast or brace immobilization, thew ankle should be placed in dorsiflexion [42].

### Kinamic simulation of external fixator adjustment for bone fracture reduction

In the management of bone fracture using an external fixator, adjustment of the bone segment is often necessary to reduce the residual deformities. For a unilateral external fixator, the ability to adjust rotational and translational deformities is limited. Furthermore, if favorable local biomechanical conditions can be reliably and conveniently implemented and maintained using an external fixator, fracture or osteotomy union can be greatly enhanced. The Dynafix^® ^(EBI, Parsippany, New Jersey) unilateral external fixator is composed of four pins inserted into the proximal and distal bone segments, two telescoping pin clamps, a central rotary joint, and four sets of revolute joints. A transverse fracture was simulated at the midshaft of the tibia and the bone segments were modeled as rigid bodies. The malalignment of the proximal segment with respect to the distal fragment, expressed by the transformation matrix, was determined radiographically using anatomical landmarks. For a 30° rotational malalignment and a 6-mm fracture gap, correcting the deformity required large rotations at the two inner revolute joints and the rotary joint [26] (Fig. 7A). Based on the same adjustment solution, different correction sequences generated different reduction paths, some of which produced bone end collisions or excessive soft tissue stretching. A simultaneous adjustment of all the joints in small increments was found to be the optimal reduction with minimal soft tissue interruption and no bony interference. This is a typical example of using VIMS technology for practical and relevant clinical application based on biomechanical analysis results.

Another example is shown to illustrate the reduction of a distal radial fracture and the corresponding fixator joint adjustments required. The same optimization process went through an average of 34 iterations to arrive within 1.e-7 of the objective function for the final solution parameters. The convergence rate averaged 3 seconds on a modern personal computer using the neutral fixator configuration as the initial solution estimate. Upper and lower bounds imposed on the revolute joints improved the solution convergence and avoided redundant solutions due to the periodicity of the trigonometric functions. For each treatment sequence for the same wrist fracture and fragment displacement, the bone correction path was calculated from the relative 3D coordinates of the fracture ends. Adjustments performed upon the joints individually resulted in the largest (32.1 mm) deflections of the distal fragment off the long axis in the axial plane whereas the incremental reduction had a maximum deviation of 4.89 mm. The axial plane deviation was calculated as the magnitude of the x and z coordinate differences of the distal fragment relative to the proximal fragment (Fig. 7B).

### Range of motion after total hip replacement

Stem-on-cup and femur-on-pelvis impingement limit the hip range of motion after total hip replacement. A computer graphic model was used to quantify the range of motion for an intact femur and after total hip arthroplasty [31] (Fig. 5). The influence of cup orientation and acetabular wear on the range of motion was quantified. The maximum flexion was similar for the intact femur and the implanted stem with the cup at 45° abduction and 10° anteversion. The maximum flexion increased as the cup was abducted and anteverted. The maximum extension was greater for the implanted stem than the intact femur for all cup orientations, with the exception of a small range with the femur externally rotated. The maximum abduction for the intact femur was greater than or equal to the maximum abduction for the implanted stem with the cup at 45° abduction and 10° anteversion and the hip rotated internally. Impingement occurred between the prosthesis neck and acetabular rim when the femur was externally rotated, but occurred between the greater trochanter and pelvis for internal rotation. The maximum abduction increased as the cup was abducted. Stem implantation had little influence on the maximum adduction because of impingement between the lesser trochanter and the pelvis. A typical superior wear pattern decreased the maximum flexion, extension, and abduction by 9° or more with the femur at 0° internal rotation.

## Model validation and scope of application

**Figure 11:**
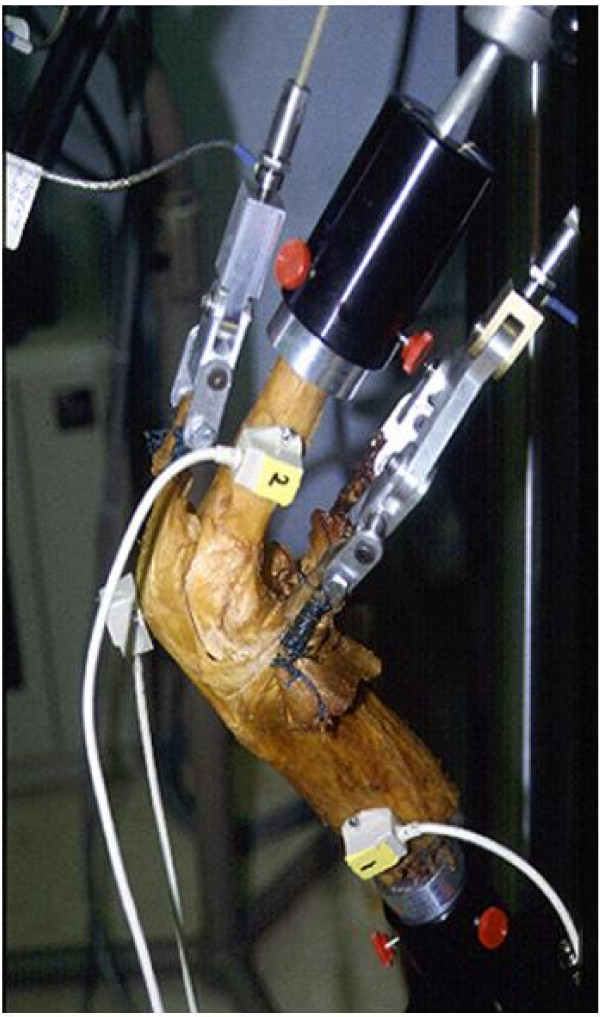
The Dynamic Knee Simulator used to study knee flexion and joint loading under simulated squatting activity. Independent loads are applied to the simulated hip joint, the medial and lateral hamstrings tendons and the quadriceps tendon using hydraulic actuators. The tendons are secured to the loading actuators using cryo-clamps. The MTS Model 790.00 TestStar™ II Control System software (MTS Systems Corporation, Eden Prairie, MN) was used to control and monitor all motion and loading conditions.

Sufficient validation of the virtual models used in VIMS technology is essential to establish its needed credibility. The Dynamic Knee Simulator (Fig. 11) developed by the author in closed collaboration with MTS (MTS System Corporation, Eden Prairie, MN) will be used to validate the model and analysis algorithms related to the knee joint [30]. On this simulator, the knee specimen is instrumented to measure a simulated squatting activity under gravitational load of the body. The measured joint contact pressure and the bone internal force and moment will be compared to that calculated using the VIMS-Tool computational algorithms and the generic knee model available in the VIMS-Model library after appropriate scaling. Absolute validation of the virtual model would be difficult but unnecessary as long as the trend of the predicted result match that measured on the Simulator under an identical loading regime. Additional test setup involving other joint models and analysis conditions will be developed to provide an overall qualitative assessment of VIMS-Model, VIMS-Tool, and VIMS-Lab.

## Discussion and summary

The "Virtual Human" is an exciting reality for biomechanical analyses and simulation well demonstrated in this paper using the musculoskeltal system as the example. With further development, this technology shall become a broad foundation with full-featured analysis capability, robust model library and database, and a well-organized laboratory environment to serve as a biomechanical simulator for a wide spectrum of basic science and clinical applications. This simulation technology unites the expertise in biomechanical analysis and graphic modeling to investigate joint and connective tissue mechanics and to visualize the results in both static and animated forms together with the system involved. Adaptable anatomical models including implants and fracture fixation devices and a computational infrastructure for static, kinematic, inverse and forward dynamic, joint contact pressure, stress and strain analyses under varying boundary and loading conditions are incorporated on a common software platform is certainly a timely and significant advance in the field of musculoskeletal biomechanics to provide the needed impetus to revive its interest and emphasis.

This simulation technology will in no way to completely replace the need to conduct experimental testing using human and animal anatomical specimens mounted on universal testing machines or custom-made joint simulators. Although time-related simulation on material fatigue failure or tissue growth and remodeling, animal study is still the main stack in bone and joint research and implant development. The results generated from all of these experimental studies, experimental or theoretical, will rely on controlled clinical trials to prove their relevance and efficacy. What the VIMS can offer is a generic database for comparative purpose for normal and patient population studies. In individual patient, it also provides the unprecedented capability to assist physician and surgeon to optimize treatment protocol to improve clinical outcome and minimize risk.

This simulation software and database were developed for the purpose of enhancing research, education, and clinical patient care related to musculoskeletal joint function at the structure, organ, and system levels. No effort is made to model and analyze connective tissue at the material level. Therefore, VIMS at its current development is limited to structural analyses of the musculoskeletal system to provide the front-end data which could be used later for the down-stream tissue level modeling and analysis purpose. It would be desirable, however, that the analysis tools for muscle force determination could include some neuromuscular control theory so that future simulation of musculoskeletal system can be expended to include synthesis problem related to its physiological performance. From the clinical point of view, this technology should have strong appeal to both patient care and rehabilitation training using its unique graphic-based models and computer animation of their biomechanical responses to loading and motion under normal and pathological conditions.

Several computational algorithms and model library database have been integrated into the VIMS software platform on a SGi super computer main frame under the Unix operating system. All of the independent analysis components of the software are accessible through a single graphical user interface (GUI). This software package could be modified to fit the X-Windows/OpenGL environment in the future. The users will get the access to the VIMS database and search through the model library to select the desirable musculoskletal region and the orthopaedic implant or device for the intended simulation and analysis. The kinematic data of the anatomic system involved in functions of daily living or sports activities could be adapted from the literature or measured to serve as the input data for biomechanical analysis on the generic models. The analysis results will be graphically presented and animated using the VisLab™ software (EAI, Ames, Iowa). Unfortunately, this utility software plus the VisModel™ package are no longer being served by the commercial firm and they need to be converted to a PC-based operating system in order for the VIMS to gain acceptance and popularity in the public domain.

The VIMS system was developed with the intention of being shared among a small group of devoted users. The current version of VIMS system is distributed among nine institutions worldwide to form the basic members of a users' organization. To assure uninhibited and unlimited utilization of the original form of the VIMS software and its analysis concept, all users are obliged to follow a set of guiding principles: 1) To follow the "Copyleft" restrictions; 2) To share the new developments in model refinements and computational algorithms; 3) To provide free consultations and trouble-shooting services among all users; and 4) To provide an "Open Door" policy to encourage surgeons and bioengineers to utilize VIMS for basic research and clinical application. A users' group, the "**VIMS.org**", will be organized to facilitate software and model library upgrading. Ultimately, Internet access option to the software should be established but to maintain the necessary security and assure a user-friendly environment would be the critical challenge. To maintain the vitality of this technology and continue to serve the general users in the field, limited patents and copyrights will be necessary to provide specific software systems in different anatomic regions for special orthopaedic applications using the Windows operating system will broaden the utility of this powerful simulation tool to revive the importance of biomechanics in musculoskeletal system reconstruction and rehabilitation.

This integrated system will no doubt making the learning of functional anatomy easier and creating the virtual laboratories on the Internet to share the resources, analysis algorithms and research findings. Such capability will expand the scope and utility of musculoskeletal biomechanics without relying upon the use of animals or cadaver specimens while restricted by the limitation of models and loading complexity. This broad-based technology will not only revolutionize the development and testing of orthopaedic implants and devices to improve their clinical performance and reliability, it will also make biomechanics competitive in landing federal funding and industrial contract. Finally, the development of biomechanically justified preoperative planning strategy and the associated execution procedures and operational steps under a virtual reality environment using accurate and realistic graphic models combined with biomechanical rationales will provide the essential foundation and tools for the true computer-aided orthopaedic surgery (CAOS). Other possibility of adapting VIMS to other medical application such as computer-aided rehabilitation (CAR) is only steps away from the reality.
